# Ultra-low-input cell-free DNA sequencing for tumor detection and characterization in a real-world pediatric brain tumor cohort

**DOI:** 10.1186/s40478-025-02024-w

**Published:** 2025-06-28

**Authors:** Tom T. Fischer, Kendra K. Maaß, Pitithat Puranachot, Markus Mieskolainen, Martin Sill, Paulina S. Schad, Stefanie Volz, Fabian Rosing, Tatjana Wedig, Nathalie Schwarz, Agnes M. E. Finster, Florian Iser, Jochen Meyer, Felix Sahm, Olli Lohi, Ahmed El Damaty, Benedikt Brors, Hannu Haapasalo, Stefan M. Pfister, Joonas Haapasalo, Kristian W. Pajtler, Kristiina Nordfors

**Affiliations:** 1 Hopp Children’s Cancer Center (KiTZ), Heidelberg, Germany; 2 Heidelberg Faculty of Medicine, Department of Pediatric Oncology, Hematology and Immunology, Heidelberg University, Heidelberg University Hospital, Heidelberg, Germany; 3 Division of Pediatric Neurooncology, German Cancer Consortium (DKTK) and German Cancer Research Center (DKFZ), Heidelberg, Germany; 4 National Center for Tumor Diseases (NCT), NCT Heidelberg, a partnership between DKFZ and Heidelberg University Hospital, Heidelberg, Germany; 5 Heidelberg Biosciences International Graduate School (HBIGS), Faculty of Biosciences, Heidelberg University, Heidelberg, Germany; 6 Princess Srisavangavadhana Faculty of Medicine, Chulabhorn Royal Academy, Bangkok, Thailand; 7 Department of Neurosurgery, Tampere University Hospital and Tampere University, Tampere, Finland; 8 Department of Pathology, Fimlab Laboratories Ltd, Tampere, Finland; 9 Clinical Cooperation Unit Neurooncology, German Cancer Consortium (DKTK) and German Cancer Research Center (DKFZ), Heidelberg, Germany; 10 Neurology and Neurooncology Program, National Center for Tumor Diseases (NCT), Heidelberg University Hospital, Heidelberg, Germany; 11 Clinical Cooperation Unit Neuropathology, German Cancer Consortium (DKTK) and German Cancer Research Center (DKFZ), Heidelberg, Germany; 12 Department of Neuropathology, Heidelberg University Hospital, Heidelberg, Germany; 13 Tampere Center for Child, Adolescent, and Maternal Health Research, Faculty of Medicine and Health Technology, Tampere University, Tampere, Finland; 14 Department of Pediatric Hematology and Oncology and Tays Cancer Center, Tampere University Hospital, Tampere, Finland; 15 Heidelberg Faculty of Medicine, Department of Neurosurgery, Heidelberg University, Heidelberg University Hospital, Heidelberg, Germany; 16 Division of Applied Bioinformatics, German Cancer Consortium (DKTK) and German Cancer Research Center (DKFZ), Heidelberg, Germany; 17 Heidelberg Faculty of Medicine, Faculty of Biosciences, Heidelberg University, Heidelberg, Germany

**Keywords:** Cerebrospinal fluid liquid biopsies, Cell-free DNA, Low-input samples, Circulating-tumor DNA, Copy number variation profiling, Low-coverage whole genome sequencing, Minimally-invasive disease monitoring, Longitudinal molecular biomarkers, Tumor evolution, Pediatric neuro-oncology

## Abstract

Molecular profiling of pediatric central nervous system (CNS) tumors has important clinical utility for guiding diagnostic and therapeutic strategies. Cell-free DNA (cfDNA) from liquid biopsies has been used for minimally invasive tumor profiling and longitudinal disease assessment in adult oncology and pediatric hematology. However, in pediatric neuro-oncology, low cfDNA yields pose a major barrier to translating these assays from bench to bedside. Here, we implemented a low-coverage whole genome sequencing (lcWGS) assay for *picogram*-level cfDNA inputs and applied it to liquid biopsies from a sizeable, population-based, cross-entity pediatric CNS tumor cohort (*n = 56* patients). Applying this protocol, cfDNA whole genome profiles were successfully acquired from all liquid biopsy samples (*n = 61/61* serum, *n = 56/56* CSF, *100%*). Based on copy number variations (CNVs), circulating-tumor DNA (ctDNA) was detected in *2/61* serum (*3%*) and in *25/56* CSF (*45%*) samples across various brain tumor entities. The integration of cfDNA results with clinical data demonstrated the utility of CSF lcWGS as a biomarker assay at diagnosis to distinguish cancerous from non-cancerous pineal region lesions (*n = 6* patients). Additionally, serial CSF assessment in *n* = *9* patients (*n = 29* CSF samples) enabled minimally invasive disease monitoring, with the added value of molecular profile availability in *n = 4/6* (*67%*) patients at relapse. Proof-of-concept data show the feasibility of serial CSF lcWGS to reveal tumor evolution, tumor heterogeneity and potential therapeutic vulnerabilities in a case of medulloblastoma and germ cell tumor. Our study underscores the clinical utility of a robust lcWGS-based liquid biopsy assay optimized for low-input samples. We identify use-cases for implementing liquid biopsies in the clinical management of pediatric CNS tumor patients and provide a strong rationale for integration into future trials.

**Supplementary Information:**

The online version contains supplementary material available at 10.1186/s40478-025-02024-w.

## Introduction

Tumors of the central nervous system (CNS) represent the most common form of pediatric solid tumors [1]. Diagnostic and therapeutic improvements through large clinical trials have increased patient survival rates over the past decades, but pediatric CNS tumors remain the leading cause of cancer-related deaths in childhood [1, 2]. Novel diagnostic pipelines, integrating histopathology and molecular results according to the 2021 WHO Classification of Tumors of the Central Nervous System, the introduction of prognostic subgroups, and the design of innovative molecularly-stratified trials are a result of recent large-scale molecular profiling studies in the field [3–9]. At present, integrated histopathological-molecular diagnoses require tumor tissues obtained via invasive procedures such as neurosurgical resection or tissue biopsy which, however, are not feasible in all cases [10, 11]. For disease monitoring, magnetic resonance imaging (MRI) is the standard-of-care approach, while cerebrospinal fluid (CSF) cytology and germ cell tumor markers are routinely assessed but have limited sensitivity [12–15]. Despite the advancements in molecular tumor profiling, effective molecular biomarkers for disease monitoring have yet to be developed in pediatric CNS cancers. Consequently, there is a high clinical need to establish novel methods for tumor detection, molecular characterization, and longitudinal disease assessment to aid in the clinical management of pediatric brain tumor patients.

Liquid biopsy is a rapidly evolving methodology that analyzes circulating tumor materials, such as cell-free DNA (cfDNA), cell-free RNA (cfRNA), extracellular vesicles, or circulating tumor cells (CTCs), in patients’ CSF, blood, or other bodily fluids [16, 17]. cfDNA is shed into body fluids during cellular turnover and is composed of DNA fragments of approximately 160 base pairs in length [18]. The fraction of cfDNA derived from tumor cells is called circulating-tumor DNA (ctDNA). In contrast to tissue biopsies, liquid biopsies can be collected minimally invasively and longitudinally across the course of the disease. In addition, liquid biopsies hold the promise to capture spatiotemporal heterogeneity for following tumor evolution and detecting subclonal populations [16, 17].

In adult oncology and pediatric leukemia, liquid biopsies have been incorporated into clinical trial protocols for minimal residual disease (MRD) detection and treatment stratification, leading to significant advancements in clinical care [19–21]. In contrast, liquid biopsy assays are still in their infancy for childhood CNS tumors. While previous studies have shown the feasibility to detect ctDNA based on genetic and genomic alterations in liquid biopsies collected from pediatric CNS tumor patients [22–29], significant hurdles remain to be overcome before liquid biopsy methodology can move from bench to bedside. Compared to adult cancers, pediatric tumors have low mutational burden and low frequency of recurrent mutations [4], limiting the applicability of targeted mutational profiling assays to tumor entities with dominant driver mutations such as high-grade gliomas [28, 30]. Given low ctDNA detection rates in blood-derived liquid biopsies from brain tumor patients, CSF has been suggested as the richest source of ctDNA in neuro-oncology [24, 31, 32]. However, the key limitation to systematic evaluation and broad application of liquid biopsy methods across pediatric CNS tumor cohorts is the small sample volume and the low cfDNA concentration typically observed in CSF samples collected in this patient population [25, 33, 34]. In this study, we established a robust wet-lab and bioinformatics workflow for genome-wide profiling of copy number variations (CNVs), which are frequently observed across pediatric CNS tumor entities [35], from trace amounts of cfDNA. This method was successfully applied across a large, population-based cohort of pediatric CNS tumors (*n = *56 patients) with banked serum (*n = *61) and CSF (*n =* 56) liquid biopsies and demonstrated utility for the clinical management of pediatric CNS tumor patients, providing a framework for its translation and prospective validation.

## Materials and methods

### Study design, eligibility, and participants

We certify that the study was performed in accordance with the ethical standards as laid down in the 1964 Declaration of Helsinki and its later amendments or comparable ethical standards. The study was approved by the Finnish Committee of Ethics of Tampere University Hospital (ethics approval ID: *R13050*). Written, informed consent was obtained from all patients and/or their legal representatives. This study collected liquid biopsies from pediatric patients (age at diagnosis < 18 years) with CNS tumors seen at Tampere University Hospital (Tampere, Finland) between 2013 and 2023. Liquid biopsies were collected for *n* = 56 patients. Exclusion criteria comprised no available tumor material for molecular studies, participation in other studies, or no available liquid biopsy (neither serum nor CSF). An integrated tumor diagnosis according to the WHO classification of CNS tumors was made at the Department of Pathology, Fimlab Laboratories, Tampere University Hospital. Clinical data, pathology work-up including CSF cytology, and radiological data were collected prior to obtaining liquid biopsy results. In addition to the tumor patient cohort, non-oncological controls (*n = *10 blood, *n* = 14 CSF controls) were collected at Heidelberg University Hospital (Heidelberg, Germany) (ethics approval ID: *S795/2020*).

### Collection of liquid biopsy samples

All liquid biopsy samples were collected as part of standard clinical care during diagnosis, staging, or follow-up (see details per sample in Table S2). Whenever possible, matched CSF and serum samples were collected at the same time point. CSF was collected in sterile tubes and stored immediately at -70 °C. Intra-operative CSF samples were collected after dura opening before touching the tumor, if possible. For serum samples, blood was drawn by standard peripheral venipuncture in BD Vacutainer tubes containing a clot activator and centrifuged at 2000 rpm for 10 min at room temperature. The supernatant was collected in sterile tubes and stored immediately at -70 °C. All liquid biopsy samples collected at Tampere University Hospital were shipped to the German Cancer Research Center (Heidelberg, Germany) on dry ice for molecular work-up.

### cfDNA isolation

Liquid biopsy samples were thawed on ice, and cfDNA was extracted using the NucleoSnap cfDNA kit (Machery Nagel, Düren, Germany) according to the manufacturer’s instructions. Elution was performed with 50 µl of nuclease-free water. The amount of total extracted DNA was quantified using the Qubit 3.0 fluorometer with the Qubit dsDNA high sensitivity assay kit (Thermo Fisher Scientific, Waltham, MA, USA). To assess fragment length distribution and quantify cfDNA concentrations, samples were subjected to automated electrophoresis on the Agilent 2100 Bioanalyzer platform adjusted to a concentration of 1 ng/µl using the High Sensitivity DNA Kit (Agilent Technologies, Santa Clara, CA, USA). The limit of detection of this assay is 5 ng/µl according to the manufacturer’s information. cfDNA concentrations in the size range of one, two, and three nucleosome peaks (approximately 160 bps and multiples thereof) as well as total DNA concentrations in the range of 50–7000 bp were quantified, using the 2100 Bioanalyzer Expert software (Agilent Technologies, Santa Clara, CA, USA).

### Low-coverage whole genome sequencing

#### Library construction

lcWGS libraries were constructed with the Accel-NGS 2 S Hyb DNA Library Kit and the Accel-NGS 2 S Set A + B MID Indexing Kit (Swift Biosciences, Ann Arbor, MI, USA) as previously described by our group [23, 27, 36]. cfDNA was used as template without prior fragmentation. If possible, the library input was adjusted to *≥ 100 pg* of cfDNA. For samples with no measurable cfDNA input, the number of amplification cycles were adapted to 12–15. No-template controls were included in each experiment. Quality control of libraries was carried out using the Qubit and the Bioanalyzer kits, as detailed above. Libraries were multiplexed in equimolar amounts at around 0.2 nM at an insert size of 50–400 bp per sample and sequenced at 100 bp paired-end on a NovaSeq 6000 (Illumina, San Diego, CA, USA) resulting in a median coverage for serum of *1.83* (range: *0.37–11.86*) and for CSF of *1.74* (range *0.21–7.61*).

#### Next-generation sequencing data preprocessing

All sequencing data were transferred to the DFKZ Omics IT and Data Management Core Facility (ODCF) and pre-processed using the in-house one-touch-pipeline *AlignmentAndQCWorkflows* (v1.2.73-1) [37]. This workflow includes adapter trimming, sequence alignment to the human reference genome (*GRCh37*, *hg19*) [38], marking duplicate reads, sorting, and extracting the sequence quality matrix. Unique molecular indexing (UMI) sequences were used for PCR deduplication using the fgbio workflow (v1.1.0; Fulcrum genomics). In brief, UMI sequence were merge into the alignment by using the matching read information using fgbio: FastqToBam and Picard MergeBamAligner [39]. The reads were grouped by the UMI RX tag and the molecular consensus reads were identified considering only those reads with mapping quality of 20. Finally, de-duplicated molecular consensus reads were subjected to Burrows-Wheeler Aligner (BWA-mem) [40]. All samples met the cut-off criterion of a genome coverage > 0.1 for further analysis.

#### Copy number variation calling and estimation of ctDNA fraction

Segmentation for copy number interrogation and estimation of the *ctDNA fraction* were performed using ichorCNA (v0.3.2) which calculates normalized log2 ratios from read count information for each genomic 1 megabase-sized window [41]. All samples met the cut-off criterion of an ichorMAD (MAD = median absolute deviation) score < 0.15 for further analysis. CNV profiles were visualized using R (v3.6.0). A Panel-of-Normal (PoN) for blood and CSF liquid biopsies was separately developed from the control samples collected from non-oncological patients. As the cfDNA of CSF may contain high amounts of ctDNA, ichorCNA default settings were adapted to allow detection of samples with small CNV segments. The parameters were modified as follows:–ploidy “c(2)”–normal “c(seq(0.5,0.8,0.1),0.85,0.9,0.95)”–maxCN 5–includeHOMD FALSE–chrTrain “c(1:18,20:22)”–estimateNormal True–estimatePloidy True–estimateScPrevalence True–minSegmentBin 20–altFracThreshold 0.05–scStates “c(1,3)”. As the majority of serum cfDNA may contain low amounts of ctDNA, ichorCNA parameters, allowing estimation of low level of ctDNA, were modified from the default values as–ploidy “c(2,3)”–normal “c(0.8,0.9,0.95,0.99,0.995)”–maxCN 4–includeHOMD True–estimateScPrevalence FALSE–scStates “c()”. Hsigh-confidence threshold for automated tumor detection of 5% and 3% for CSF and serum, respectively, were established.

To facilitate the comparison between tumor and cfDNA CNVs, ichorCNA detected CNVs based on sequencing coverage segmentation per 1-megabase of genomic non-overlapping windows. Based on the segmentation, the software detected copy number aberrations by fitting a model with a range of parameters for tumor fractions (TF) and tumor ploidy. Multiple solutions of CNV profiles were reported but only the profile with the highest likelihood score was considered. A tumor CNV segment was defined as detected in cfDNA when the majority of the overlapping cfDNA segment reported the same CNV event (AMP or DEL). Taking into account the estimated ctDNA fraction and the visual comparison between tumor and copy number changes, liquid biopsy samples were annotated as “ctDNA positive” or “ctDNA negative”.

#### Estimation of longitudinal clonal tumor evolution

Intratumor heterogeneity and longitudinal clonal evolution were reconstructed using the Canopy package [42]. Matched whole-exome sequencing serial tumor and germline data for patient #40 was produced and kindly provided by the INdividualized Therapy FOr Relapsed Malignancies in Childhood (INFORM) program, with approval by the national coordinator (local ethics approval: *R17003*) [9, 43–45]. Somatic functional SNV/Indels were called for the tumor and interrogated from matching CSF lcWGS by considering read pileup information. Tumor clonal structure is estimated based on the configuration with the highest posterior likelihood.

### DNA methylation-based tumor classification

Established protocols for DNA methylation profiling based on bisulfite conversion were used, applying the EZ DNA Methylation Kit (Zymo, Irvine, CA, USA) and the Infinium HumanMethylation450, MethylationEPIC v1, and MethylationEPIC v2 (Illumina, San Diego, CA, USA) [3]. Methylation-based tumor classifications were obtained using the Heidelberg Brain Tumor Classifier *v12.5*; except for tumor samples processed using the EPIC v2 chip where *v12.8* was used for classification [3]. The ‘noob’ algorithm from the minfi package was used for data preprocessing of methylation data. The conumee Bioconductor package was used to infer genome-wide CNVs and segmentation [46]. For CNV calling, we established a threshold for calling amplification (3 copies or more) and deletion (1 copy or less) to 0.07 and − 0.07, respectively.

### Statistical analysis and data visualization

Patient data were managed and analyzed in a pseudonymized form. Given heterogeneous liquid biopsy sampling time points, the clinical and molecular data available for the most recent tumor episode was used for annotation of liquid biopsies with clinical and tumor information, ranging from pre-operative sampling within two weeks before surgery to sampling during follow-up up to 5.8 years since the last tumor resection. Data management and statistical calculations were performed using PRISM Statistical Software 9 (Graphpad, La Jolla, CA, USA), Excel (Microsoft, Redmond, WA, USA), and R *v3.6.0*. Mann-Whitney U and Kruskal-Wallis test followed by Dunn’s multiple comparison test were used for continuous variables of two groups or multiple groups, respectively. Fisher’s exact test was used to investigate liquid biopsy results in association with clinical and molecular annotations. *p*-values < 0.05 were considered statistically significant and the following notations were displayed in the plots: * *p* < 0.05, ** *p* < 0.01, *** *p* < 0.001, **** *p* < 0.0001. If not stated otherwise, box plots show median with interquartile range, whiskers from minimum to maximum, and all data points. Receiver operating characteristic curve (ROC) analyses were performed using the precrec and pROC R packages [47, 48]. ComplexHeatmap [49] was used for the visualization of potentially actionable CNVs and genes based on filtering for the INFORM and neuropathology gene panel list of reportable alterations [7, 9, 43–45]. The Integrative Genomics Viewer (IGV) was used to visualize genome-wide CNV profiles [50]. Graphical illustrations were created with *BioRender.com*.

## Results

### Liquid biopsy collection in a real-world pediatric CNS tumor cohort

**Fig. 1:**
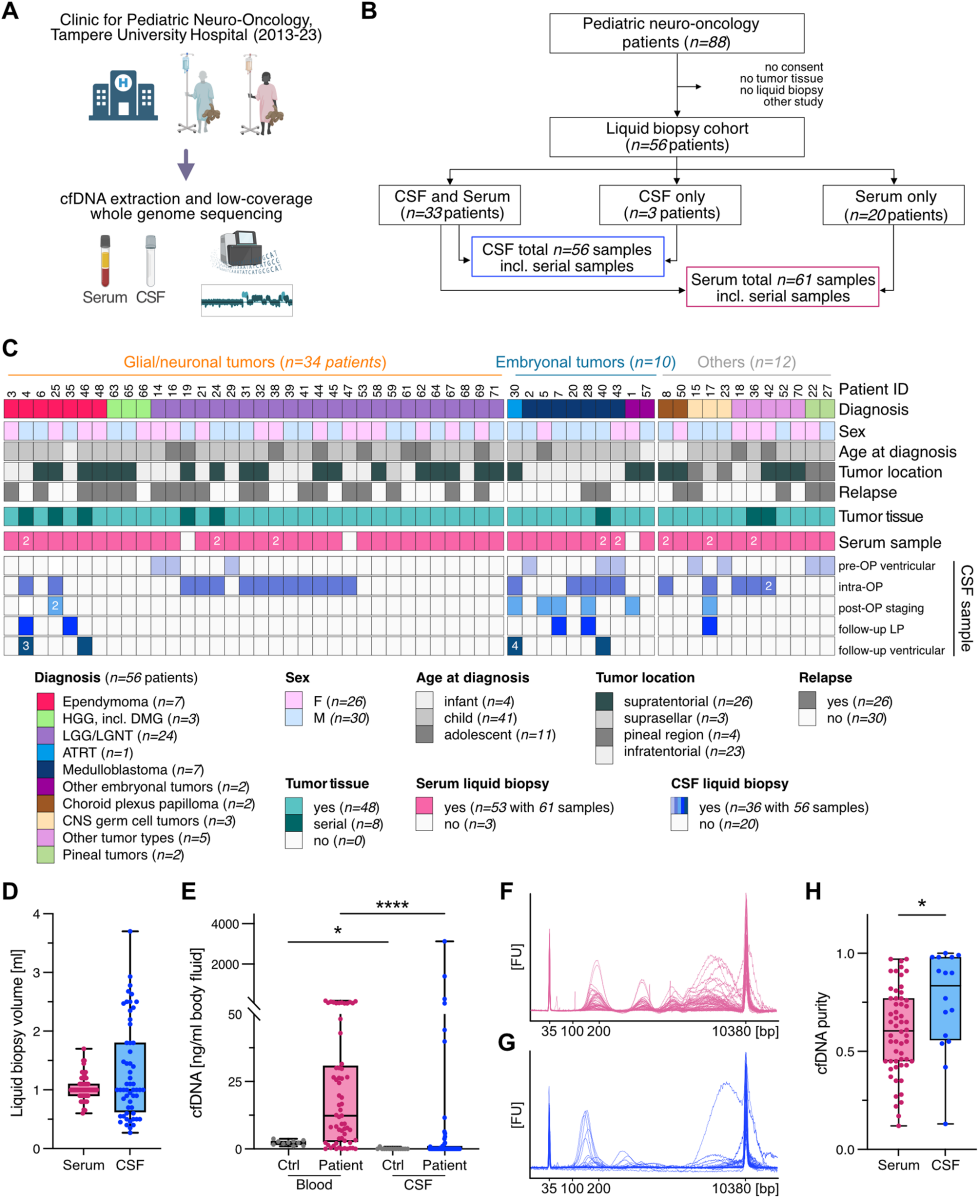
Overview of the pediatric neuro-oncology liquid biopsy cohort. (**A**) Schematic illustration of the study workflow. (**B**) Consort diagram delineating the composition of the study cohort and the liquid biopsy sample distribution. (**C**) Summary of the liquid biopsy study cohort (*n = *56 cases), annotated with clinical information, biomaterials, and analyses performed. Numbers in cells denote sample size if > 1 liquid biopsy per sample type. (**D**) Available liquid biopsy volumes in *ml*. *n = *61 serum (median = 1); *n* = 56 CSF (median = 1). (**E**) cfDNA concentration in *ng/ml* body fluid, *n* = 10 ctrl blood (median = 2.34); *n* = 61 patient blood (* median = *12.37); *n* = 14 ctrl CSF (* median = *0), *n* = 56 patient CSF (* median = *0). (**F-G**) Size distribution profiles of serum cfDNA and CSF cfDNA samples, respectively. (**H**) cfDNA purity quantified as cfDNA (1.-3. peak) over total DNA concentration (50–7000 bp) per sample, *n* = 56 serum, *n* = 16 CSF, samples with no measurable cfDNA excluded

Between 2013 and 2023, *n* = 88 pediatric CNS tumor patients were seen at Tampere Children’s Hospital, Tampere, Finland. To assess feasibility and clinical utility of liquid biopsies in this real-world, unselected patient population, liquid biopsy samples were banked during routine clinical procedures for subsequent molecular profiling for *n* = 56/88 patients (64%) (Fig. 1A-B). Serum samples were available for 53/56 (95%) patients, including *n* = 45 patients with unique and *n* = 8 patients with *n* = 2 serum samples, for a total number of *n* = 61 serum samples. Serum samples were collected pre-operatively (*n* = 41 serum samples), post-operatively (*n* = 17), and during follow-up (*n* = 3). CSF samples were available for 36/56 (64%) patients, including *n* = 27 patients with unique and *n* = 9 patients with serial CSF samples (range: *n* = 2–6 samples per patient), for a total number of *n* = 56 CSF samples (Fig. 1B). Patient-matched CSF and serum samples were collected in *n* = 33 patients (Fig. 1B). CSF samples were collected pre-operatively via ventricular access (*n* = 10 CSF samples), intra-operatively (*n* = 24), during post-operative staging (*n* = 8), and during follow-up via lumbar puncture (*n* = 5) or ventricular sampling (*n* = 9) (Fig. 1C, Table S1-S2). Demographic, clinical, and diagnostic information are summarized per patient in Fig. 1C and Table S1. For the current study, cfDNA extraction was performed in the laboratory as previously established [23]. Comparable sample volumes were available for serum (median: 1 ml, range: 0.6–1.7) and CSF (median: 1 ml, range: 0.3–3.7) (Fig. 1D). cfDNA was measurable in 59/61 (97%) serum samples with a median concentration in the *nanogram* range (median: 12.4 ng/ml serum, range: 0-177.7 ng/ml) *(*Fig. 1E-F*).* In contrast, cfDNA concentrations were below the limit of detection in the *picogram* range for 40/56 (71%) CSF samples, with only 16/56 (29%) CSF samples yielding measurable cfDNA amounts (median: 6.44 ng/ml CSF, range: 0.42-3125.4 ng/ml) *(*Fig. 1E and G*).* Fragment length distribution analysis showed a significantly higher purity of cfDNA in CSF compared to serum (median serum vs. CSF: 61% vs. 84%, *p* = 0.01) (Fig. 1H), corroborating the concept of a lower background signal in CSF compared to blood [51]. These pre-analytical results underscore the challenges of applying liquid biopsy assays across pediatric CNS tumor cohorts without introducing cohort bias due to the reliance on high cfDNA inputs, prompting the development of a robust and more adaptable method.

### lcWGS-based CNV profiling from minimal amounts of cfDNA

**Fig. 2:**
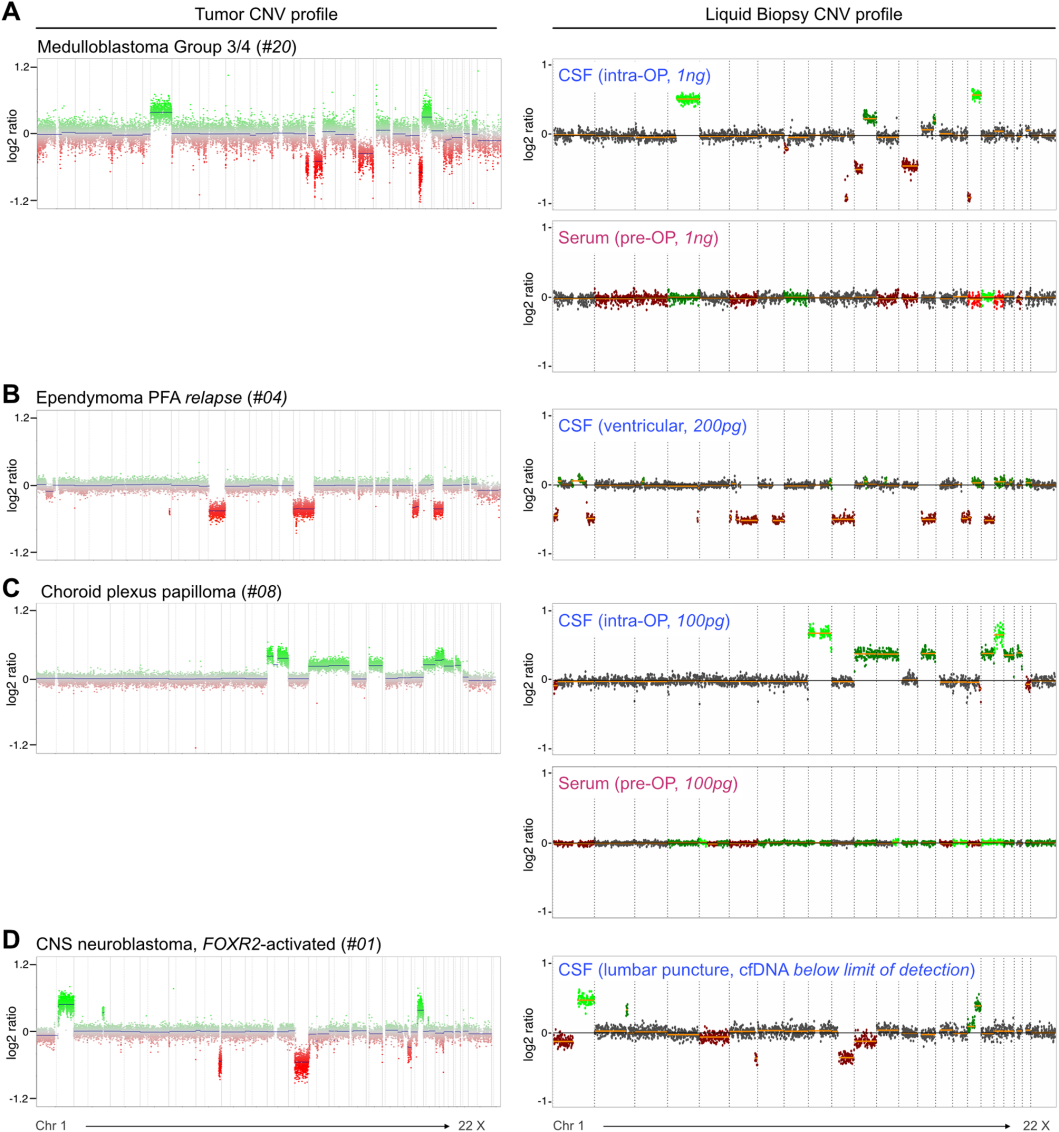
lcWGS-based copy number profiling from minimal amounts of cell-free DNA. (**A-D**) Examples of lcWGS-derived CNV profiles for cfDNA input amounts on a range from *nanograms* to *picograms* demonstrating robust assay performance. (**A**) Patient #20 diagnosed with medulloblastoma group 3/4. CSF: collected intra-operatively, 1 ng of cfDNA used for library preparation. Serum: collected prior to surgery, 1 ng of cfDNA used for library preparation. (**B**) Patient #04 diagnosed with relapsed ependymoma PFA. CSF: collected via ventricular shunt during follow-up. 200 pg of cfDNA used for library preparation. (**C**) Patient #08 diagnosed with choroid plexus papilloma. CSF: collected intra-operatively, 100 pg of cfDNA used for library preparation. Serum: collected prior to surgery, 100 pg of cfDNA used for library preparation. (**D**) Patient #01 diagnosed with CNS neuroblastoma, *FOXR2*-activated. CSF: collected via lumbar puncture during post-operative staging. The cfDNA concentration was below the limit of detection, 10 µl of the 50 µl cfDNA isolate were used for library preparation

**Fig. 3:**
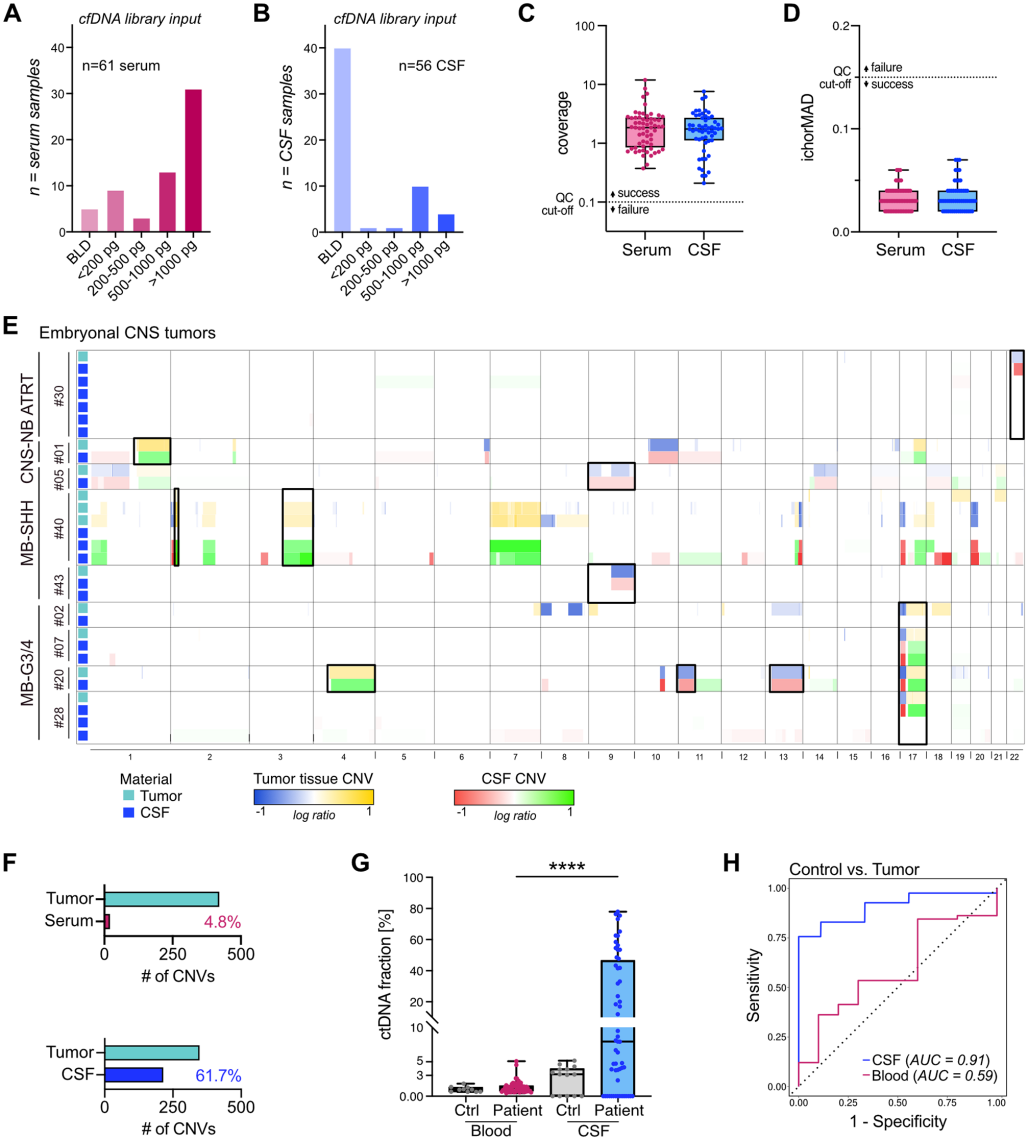
lcWGS-based tumor detection in pediatric CNS tumor liquid biopsies. (**A-B**) Bar graphs depicting cfDNA input amounts used for lcWGS library preparation for serum (**A**) and CSF (**B**) samples. BLD = below limit of detection. (**C**) Box plot showing sequencing coverage per sample, *n* = 61 serum (median = 1.83), *n* = 56 CSF (median = 1.74). All samples (117/117, 100%) passed the quality control threshold for a minimum coverage of > 0.1x. Y-axis was log10 transformed. (**D**) Box plot showing ichorMAD score per sample, *n* = 61 serum (median = 0.03), *n* = 56 CSF (median = 0.03). All samples (117/117, 100%) passed the quality control threshold for an ichorMAD score of < 0.15. MAD = median absolute deviation. (**E**) Genome-wide CNV profiles of CSF and matched tumor tissue samples for embryonal CNS tumors. Hallmark cytogenic events are highlighted with black boxes. CNS-NB = CNS neuroblastoma, *FOXR2*-activated. (**F**) Detection rate of tumor-derived CNVs in liquid biopsies across the study cohort: serum 20/419 (4.8%), CSF 214/347 (61.7%). In 9 of 56 patients, the tumor did not show CNVs. (**G**) Calculated ctDNA fraction in [%], *n* = 10 ctrl blood (median = 1%); *n* = 61 patient blood (median = 1%); *n* = 14 ctrl CSF (median = 3%), *n* = 56 patient CSF (median = 8%). (**H**) ROC curve for detecting tumor vs. control samples with a ctDNA fraction cut-off of 3% for serum and 5% for CSF. Only including samples where matched tumor has CNVs and liquid biopsy ctDNA fraction is > 0. *n* = 9 ctrl CSF, *n* = 41 patient CSF, *n* = 10 ctrl blood, *n* = 58 patient blood. ROC = receiver operating characteristic

To investigate detectability of circulating-tumor DNA (ctDNA) based on CNVs from minute amounts of cfDNA, we established an optimized protocol for cfDNA-compatible lcWGS library preparation [23, 27]. This assay allowed for CNV profiling with library input requirements as low as *picograms* of cfDNA, including cfDNA concentrations below the limit of detection (Fig. 2A-D). Across the whole cohort, the majority of libraries was generated from *picograms* of cfDNA (Fig. 3A-B). Our assay successfully generated WGS data for all 117/117 (100%) liquid biopsy samples, meeting the set quality control cutoffs of > 0.1 coverage and < 0.15 ichorMAD score (median coverage serum: 1.83, CSF: 1.74, Fig. 3C-D, Table S2). Systematic comparison of lcWGS-derived and matched tumor tissue-derived CNV profiles demonstrated detection of tumor-derived CNVs as readout of ctDNA in liquid biopsies across entities (Fig. 3E-F, S1). Of all copy number alterations detected in matched tumors, excluding copy number neutral cases (7/34 glioneuronal, 2/12 other tumors), 4.8% and 61.7% of CNVs were detected in serum and CSF samples, respectively (Fig. 3F). For embryonal tumors, where all tumor methylation arrays showed CNVs, ctDNA was detected based on CNVs in the CSF of 8/9 (89%) patients (Fig. 3E). Hallmark cytogenetic events such as loss of chromosome (chr) 22 in atypical teratoid/rhabdoid tumor (ATRT) (case #30), chr1q gain in CNS neuroblastoma with *FOXR2*-activation (#01), chr9q deletion in sonic hedgehog (SHH)-activated medulloblastoma (MB) (#5, #43), *MYCN* amplification in relapsed MB-SHH (#40), and i(17q) in MB Group3/4 (G3/4) (#7, #20, #28) were readily detected in CSF (Fig. 3E) [52–54]. CNVs were detectable in the CSF for 5/18 (28%) patients with glial/neuronal tumors and 6/9 (67%) patients with other tumor types (Fig. S1).

Calculating the fraction of ctDNA in the pool of cfDNA based on copy number abnormalities, CSF samples showed significantly higher ctDNA fractions (median: 8%, range: 0–78%) compared to serum (median: 1%, range: 0–5%) (Fig. 3G). CSF samples, with a ctDNA fraction cut-off at 5%, provided an accuracy of 0.76 for positive tumor detection, and serum samples, with a ctDNA fraction cut-off at 3%, an accuracy of 0.16. Overall, *n* = 31 CSF patient samples were *ctDNA negative* and *n* = 25 were *ctDNA positive*, based on calculated ctDNA fractions and visual review of the CNV profiles (Table 1, S2). Serum samples were annotated as *ctDNA negative* (*n* = 59) vs. *ctDNA positive* (*n* = 2), with the positive samples collected pre-operatively from patient #15 diagnosed with germinoma and patient #22 diagnosed with pineoblastoma (Table S2). For cases with CNV-positive tumor methylation arrays and liquid biopsies with ctDNA fractions greater than 0, CSF showed an AUC of 0.91 and serum an AUC of 0.59 for discriminating patient from control samples (Fig. 3H).

Taken together, these results demonstrate the reliability of our liquid biopsy lcWGS pipeline with a technical success rate of 100% across our cohort regardless of cfDNA input amounts with promising results for CNV-based tumor detection in CSF, but not serum.

### Clinical applicability of CSF cfDNA sequencing

Clinico-molecular features were compared between ctDNA negative (*n* = 31/56, 55%) and ctDNA positive (*n* = 25/56, 45%) CSF samples to contextualize the lcWGS data set (Table 1, S2). ctDNA was detected in at least one CSF sample for 19/36 (53%) patients. ctDNA detectability was significantly associated with tumor entity (Fisher’s exact *p* = 0.02), WHO grade of the primary diagnosis (*p = *0.05), *Ki-67* of the tumor (*p = *0.03), and detectability of measurable cfDNA after extraction from CSF (*p = *0.001). The significant correlation between Ki-67 positivity in tumor tissue and CSF positivity indicates that proliferative rates increased ctDNA positivity.

In comparing ctDNA detection rates between tumor entities (Fig. S2A), the highest detection rates were observed in entities where all matched tumor samples showed CNVs, including medulloblastoma (ctDNA positive: 6/7 patients, 86%; 8/13 CSF samples, 62%) and CNS germ cell tumors (ctDNA positive: 3/3 patients, 100%; 5/5 CSF samples, 100%). In contrast, only 3/14 (21%) low-grade glioma (LGG)/ low-grade neuroepithelial tumor (LGNT) CSF samples showed detectable ctDNA. Importantly, 6/14 (43%) LGG/LGNT cases were identified as copy number neutral based on tumor methylation array results.

Among other variables, tumor touching the ventricle on MRI (*p = *0.1) and hydrocephalus (*p = *0.05) at the time of CSF sampling showed a trend towards higher likelihood of ctDNA detection. In this cohort, disease episode, tumor location, M-status, and the CSF collection method was not found to significantly impact ctDNA estimations, suggesting applicability in different clinical settings. However, the small sample sizes, for example, for each collection method, warrant larger numbers for more robust statistical comparisons.

If cfDNA was measurable in pre-analytical assessments, 13/16 (81%) CSF samples were found ctDNA positive. Notably, ctDNA was detectable in 12/40 (30%) samples that pre-analytically did not yield detectable cfDNA amounts, underscoring the high clinical need for advanced methods without reliance on high cfDNA amounts. CSF cytology, which is standard-of-care for disease assessment in pediatric neuro-oncology, was positive in 5/32 (15%) assessed CSF samples. 4 of these 5 (80%) samples were ctDNA positive by lcWGS, while the negative sample was the only of 5 samples that lacked measurable cfDNA and that originated from a glial/neuronal tumor. In contrast, lcWGS detected ctDNA in 15/27 (56%) of cytology-negative samples, suggesting enhanced sensitivity for disease detection through combined cytologic and molecular CSF pathology.

In summary, these data demonstrate the robust applicability of the lcWGS assay to CSF samples in a cohort with diverse clinico-molecular characteristics. Moreover, 18/25 (72%) samples positive by lcWGS compared to 8/31 (26%) samples negative by lcWGS were collected in patients with subsequent disease progression (Fig. S2B-C), suggesting that cfDNA-derived CNVs may serve as prognostic biomarker.

**Table 1:** Association between lcWGS-based CNV profiling results for CSF and clinico-molecular features. Fisher’s exact P is shown for each investigated feature

	CSF ctDNA neg *n* = 31 (55%)	CSF ctDNA pos *n* = 25 (45%)	*p*-value
**Number of patients**	17 (47)	19 (53)	
**Diagnosis group n (%)**			*0.11*
Glial/neuronal	17 (71)	7 (29)	
Embryonal	10 (50)	10 (50)	
Others	4 (33)	8 (67)	
**Tumor Entity (if > 1 patient) n (%)**			*0.022*
Ependymoma	6 (60)	4 (40)	
LGG/LGNT	11 (79)	3 (21)	
Medulloblastoma	5 (38)	8 (62)	
CNS germ cell tumor	0 (0)	5 (100)	
Others	9 (64)	5 (36)	
**WHO grade of primary tumor n (%)**			*0.048*
I-II	15 (75)	5 (25)	
III-IV	16 (44)	20 (56)	
**Disease episode n (%)**			*0.421*
Primary	16 (50)	16 (50)	
Relapse	15 (62)	9 (38)	
**Tumor location of current episode n (%)**			*0.153*
Supratentorial	13 (62)	8 (38)	
Suprasellar	0 (0)	2 (100)	
Pineal region	1 (20)	4 (80)	
Infratentorial	16 (64)	9 (36)	
Spinal	1 (33)	2 (67)	
**M status at current episode n (%)**			*1*
M0	23 (55)	19 (45)	
M+	8 (57)	6 (43)	
Median Ki-67% of current tumor * n (%)			*0.028*
Low	16 (76)	5 (24)	
High	8 (40)	12 (60)	
**CNV status of current tumor n (%)**			*0.167*
CNN	7 (78)	2 (22)	
CNV	24 (51)	23 (49)	
**Tumor touching the ventricle on MRI n (%)**			*0.11*
No	17 (68)	8 (32)	
Yes	14 (45)	17 (55)	
**Hydrocephalus at time of CSF collection n (%)**			*0.054*
No	24 (65)	13 (35)	
Yes	7 (37)	12 (63)	
**CSF collection timing and method n (%)**			*0.901*
Pre-OP (ventricular sampling)	5 (50)	5 (50)	
Intra-OP	15 (62)	9 (38)	
Post-OP staging (lumbar puncture / Rickham)	4 (50)	4 (50)	
Follow-up (lumbar puncture)	3 (60)	2 (40)	
Follow-up (ventricular sampling)	4 (44)	5 (56)	
CSF cytology ** n (%)			*0.625*
Negative	12 (44)	15 (56)	
Positive	1 (20)	4 (80)	
**cfDNA detectable in pre-analytics n (%)**			*0.001*
No	28 (70)	12 (30)	
Yes	3 (19)	13 (81)	

* split by median 17.5%; n.a. for 15 samples, 7 CSF ctDNA neg, 8 CSF ctDNA pos

** n.a. for 24 samples, 18 CSF ctDNA neg, 6 CSF ctDNA pos

### Use-cases for CSF lcWGS assays in clinical practice

Next, we focused on three categories where CSF cfDNA profiling may have a significant impact on clinical decision making as *(i)* tumor biomarker at diagnosis, *(ii)* longitudinal biomarker for disease assessment, and *(iii)* molecular readout of tumor evolution.

#### cfDNA-derived CNVs as tumor biomarker for CNS malignancies

**Fig. 4:**
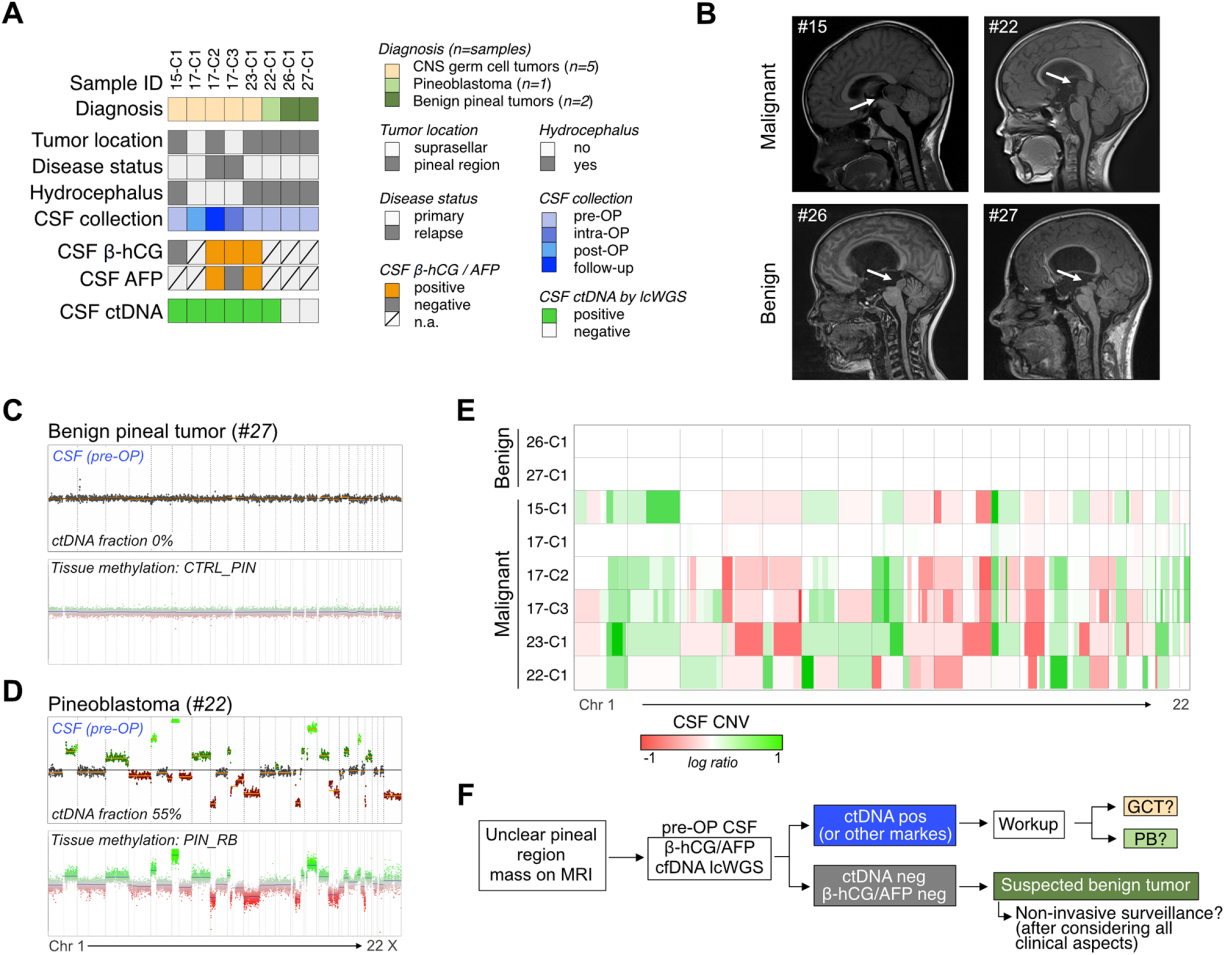
Diagnostic utility of CSF liquid biopsies in pineal region tumors. (**A**) Oncoprint summarizing cohort of patients with pineal region involvement (*n = *6 patients, *n* = 8 CSF samples). ctDNA positivity showed high biomarker accuracy in differentiating benign from malignant pineal region tumors. (**B**) Examples of MRI scans for two malignant pineal masses (case #15 germinoma, #22 pineoblastoma) and two benign pineal masses (case #26, #27). (**C**) CNV profiles derived from CSF and tissue biopsy for patient #27, diagnosed with a benign pineal region tumor. CSF collected pre-operatively from the third ventricle. (**D**) CNV profiles derived from CSF and resected tissue for patient #22, diagnosed with pineoblastoma. CSF collected pre-operatively from the third ventricle. (**E**) Genome-wide CNV profiles of CSF for all cases with pineal region involvement. (**F**) Schematic flowchart delineating potential implementation of cfDNA lcWGS into diagnostic work-up for pineal region masses. Abbreviations: AFP = alpha-1 fetoprotein, β-HCG = beta human chorionic gonadotropin, GCT = germ cell tumor, PB = pineoblastoma

Differentiating between non-cancerous and cancerous processes is a common challenge when a brain lesion is identified on MRI in children [55–58]. Unclear pineal region masses include benign tumors such as pineocytoma and malignant tumors such as pineoblastoma and CNS germ cell tumors (GCT) [59–61]. Pre-operative CSF diversion, often performed to relieve obstructive hydrocephalus and assess GCT markers (β-hCG, AFP), provides a unique opportunity to integrate cfDNA sequencing into diagnostics, with CNVs as a potential biomarker due to the high frequency of CNVs reported in pineoblastoma and CNS GCTs [62, 63], and their high specificity for malignancy. 6/6 (100%) CSF samples collected from patients with malignant tumors (*n* = 3 patients with CNS GCT, *n* = 1 pineoblastoma) were ctDNA positive by CNV profiling, clearly confirming malignancy (Fig. 4A-E). 2/2 (100%) CSF samples from two patients with benign tumors were ctDNA negative (Fig. 4A-E). While such a result cannot rule out a malignant process, it can be considered alongside standard-of-care biomarkers (β-hCG, AFP) and clinical information to support short-interval surveillance using MRI and liquid biopsies instead of invasive tissue biopsy or resection (Fig. 4F). Retrospective application of this strategy suggests that cfDNA results would have supported surveillance without tissue biopsy in patient #26, but may have spared patient #27 from an invasive biopsy of the pineal gland (Fig. 4C-F).

Taken together, CSF-derived CNVs may have utility as a specific tumor biomarker, for example in distinguishing between benign and malignant pineal region involvement in our cohort (Fig. 4E).

#### Longitudinal molecular disease monitoring enabled by CSF liquid biopsies

While standard-of-care longitudinal disease assessment in pediatric neuro-oncology includes MRI and, for high-risk cases, CSF cytology, molecular biomarkers are currently lacking. In our cohort, serial CSF samples were available in *n* = 9 cases, totaling *n* = 29 samples, including collections at early time points, i.e. pre-, intra-, and post-OP, and during follow-up via lumbar puncture or ventricular aspiration.

In the subset of *n* = 15 CSF samples that were collected during periods of abnormal MRI findings and assessed by cytology, 3/15 (20%) samples showed abnormal cytology. In contrast, of these 15 samples, 9 (60%) were ctDNA positive by lcWGS, including 2/3 cytology-positive samples and 7/12 cytology-negative samples.

**Fig. 5:**
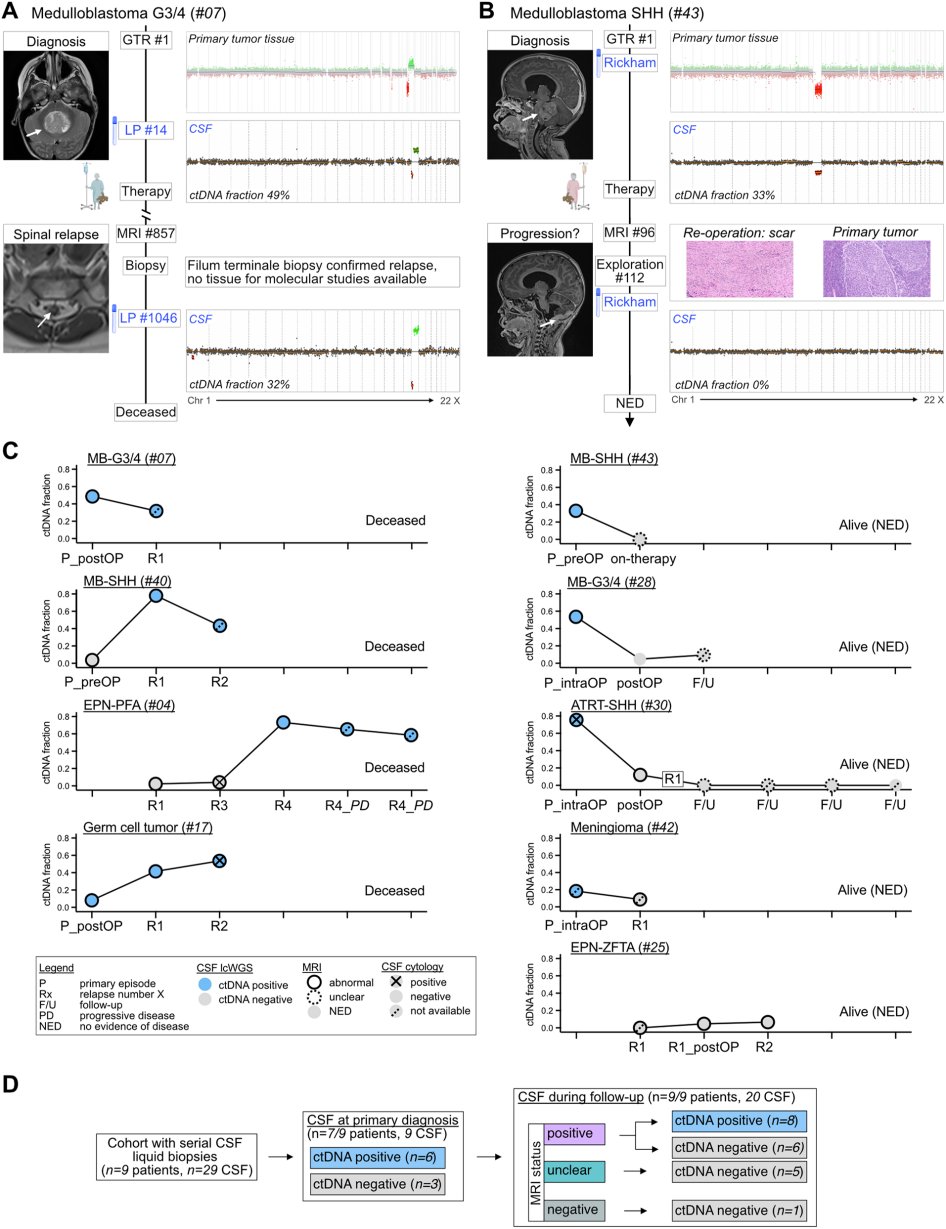
Minimally invasive surveillance strategy via longitudinal CSF liquid biopsies. (**A-B**) Clinical timelines alongside CNV profiles, #X in timeline indicates number of days since diagnosis. (**A**) For patient #07, diagnosed with relapsed MB-G3/4, CSF during follow-up shows molecular relapse. (**B**) For patient #43, diagnosed with MB-SHH, CSF collected during suspected progression based on MRI was negative. (**C**) Serial trajectories of CSF ctDNA status, annotated by clinical time points, MRI results, and CSF cytology. Follow-up times for patients with no evidence of disease: #43: >2 years, #28: >1 year, #30: 9 months, #42: >1 year, #25: >1 year. (**D**) Schematic highlighting clinical utility of CSF-derived cfDNA as a biomarker, complementing MRI-based disease assessment. *Abbreviations*: EVD = external ventricular drain, GTR = gross total resection, LP = lumbar puncture, Rickham = ventricular reservoir

The presence of CNVs in CSF samples, along with ctDNA trajectory patterns, closely reflected disease progression (Fig. 5A-C). At early time points, 6/7 cases (86%) and 6/9 samples (67%) were ctDNA positive (Fig. 5C-D). During follow-up, *n* = 20 CSF samples from *n* = 9 cases were collected at timepoints positive (14/20, 70%), unclear (5/20, 25%), or negative (1/20, 5%) by MRI. In samples with positive MRI findings, 8/14 (57%) samples were ctDNA positive, enabling disease confirmation and acquisition of molecular tumor profiles through CSF draws (Fig. 5D). For example, in patient #07, relapse of MB-G3/4 was confirmed by histo-pathological assessment of a filum terminale biopsy while the CSF liquid biopsy molecularly confirmed the relapse (Fig. 5A). In cases with unclear MRI results, 5/5 (100%) CSF samples were negative, and all three patients remained negative during further follow-up (median follow-up: 19 months, range 9–26 months). In case #43, diagnosed with MB-SHH, ctDNA negativity during ambiguous imaging could have supported surveillance over surgical intervention (Fig. 5B). In case #30, diagnosed with relapsed ATRT, *n* = 4 CSF samples taken during maintenance therapy were ctDNA negative, while MRI findings were inconclusive (treatment-related changes vs. disease progression) at three time points before eventually becoming negative at the last time point (Fig. 5C).

Taken together, lcWGS-based cfDNA profiling shows promise as a powerful, minimally invasive methodology for serial disease assessment in different pediatric CNS tumor entities.

#### Revealing tumor evolution through CSF liquid biopsies

**Fig. 6:**
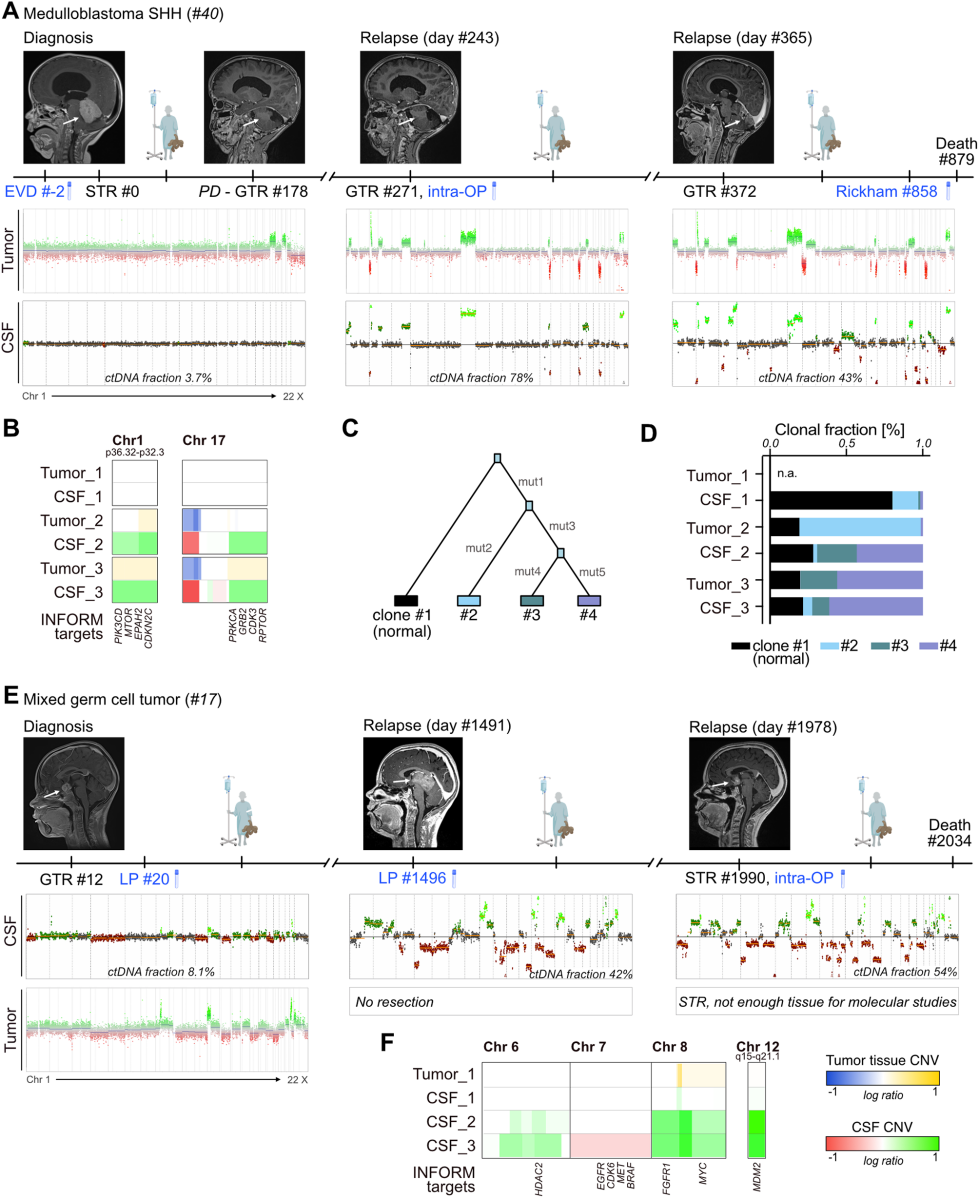
Assessment of tumor evolution using CSF-derived lcWGS profiles. (**A**) Clinical timeline alongside CNV profiles for patient #40 diagnosed with relapsed MB-SHH. (**B**) CNVs on *Chr. 1* and *17* in matched tumor and CSF profiles displaying temporal evolution that was detected earlier in CSF than tissue (patient #40). (**C**) Clonal tree derived from Canopy analysis based on SNV and indel analysis from tumor, germline, and cfDNA CSF sequencing (patient #40). (**D**) Clonal composition for matched tumor and CSF samples displaying clonal evolution identified earlier in CSF than tissue (patient #40). (**E**) Clinical timeline combined with CNV profiles for patient #17 diagnosed with relapsed mixed GCT. (**F**) Evolution of broad and focal CNVs on indicated chromosomes and at indicated gene loci in tumor tissue and CSF over the course of the disease (patient #17). *Abbreviations*: Chr = chromosome, EVD = external ventricular drain, LP = lumbar puncture

To investigate the possibility of tracking tumor evolution in serial CSF samples by lcWGS, we focused on patient #40, diagnosed with MB-SHH (Fig. 6A), as matched serial tumor and germline data were available through the INFORM registry [9]. Longitudinal CNV profiles displayed evolution in both tumor and CSF samples, including the emergence of high-risk molecular features such as *GLI2* amplification (Fig. 6A) [64]. Notably, cfDNA profiling identified CSF-exclusive CNVs at first relapse, such as chr1p or chr17q gain, that were detected in the tumor at the subsequent time point (Fig. 6B). Through SNV and indel analysis [42], deconstruction of clonal architecture allowed to track three tumor clones in tissue and CSF samples (Fig. 6C). While clone #2 was dominant in the tumor tissue at time point 2, clones #3 and #4 were already prominently represented in the matched CSF, preceding their subsequent expansion in tumor tissue at time point 3 (Fig. 6D).

For patient #17, diagnosed with mixed GCT, tumor tissue was unavailable for molecular studies at both relapse episodes (Fig. 6E). CSF collected at relapse episodes (via lumbar puncture and intra-operatively) uniquely enabled molecular profiling which demonstrated evolving CNVs, exemplified by divergent copy number status on chr6, chr7, and chr8 and a focal amplification involving *MDM2* on chr12q over the course of the disease (Fig. 6E-F). Emerging CNVs were annotated with clinically reported, potentially actionable targets from INFORM catalogue [9], suggesting that lcWGS of CSF-derived cfDNA may facilitate the identification of therapeutic vulnerabilities (Fig. 6B and Fig. S3).

Taken together, these proof-of-concept results suggest that early LB-based detection of tumor evolution and potential therapeutic vulnerabilities is feasible, warranting further validation in future studies.

## Discussion

Liquid biopsies have substantially transformed the clinical management of several adult tumors and pediatric leukemias [16, 17]. However, liquid biopsy-based molecular biomarkers for disease detection, therapy response assessment, and longitudinal disease monitoring are not yet widely available for pediatric CNS tumor patients. The low cfDNA amounts isolated from CSF of these patients compared to adult oncology [65], constitute a major roadblock for the broad deployment of previously published liquid biopsy assays. Herein, we report application of a sensitive lcWGS assay without input limitations in a sizeable, cross-entity pediatric brain tumor cohort collected during routine clinical care. Through review of the lcWGS results with detailed clinical data by a multidisciplinary clinical team, we identify use-cases for unlocking the potential of liquid biopsies and preparing their clinical translation in pediatric neuro-oncology.

In the real-world clinical setting, differences in liquid biopsy collection created challenges in harmonizing CSF banking across different brain tumor entities. For example, pre-operative CSF was collected from patients with hydrocephalus and pineal masses, whereas immediate resection precluded pre-operative sampling in medulloblastoma patients. While lumbar punctures are part of medulloblastoma trial protocols during staging and follow-up, they are not routinely performed for non-metastatic LGG/LGNTs. This led to a heterogeneously sampled CSF cohort; however, it reflects a real-world, non-trial-based clinical setting and demonstrates the robustness of cfDNA sequencing while awaiting prospective investigation of gold-standard methods for sample collections. Expanding on our previous collaborative efforts to facilitate cfDNA sequencing from trace DNA amounts [23, 27], our lcWGS workflow enabled cfDNA analysis in this sizeable, real-world cohort with all samples passing quality control metrics (i.e. >0.1x coverage), validating the robustness of this technique and applicability across different clinical scenarios. Notably, even without input restrictions, our CNV assay demonstrated similar or higher success rates compared to previous studies applying lcWGS to *nanograms* of CSF-derived cfDNA in cross-entity pediatric CNS tumor cohorts [24, 25]. Tumor entity and pre-analytical cfDNA detectability were significantly associated with ctDNA detection, while additional factors influencing liquid biopsy detection rates– such as M-status, tumor location, or anatomical sampling site reported in other studies– require investigation in larger patient cohorts [26, 27, 66]. Similarly, future studies are needed to validate the potential of ctDNA as prognostic biomarker.

lcWGS-based CNV profiling of CSF offers a valuable, impactful approach to distinguish between cancerous and non-cancerous lesions when finding unclear masses on MRI, a common challenge in pediatric neuro-oncology [55–58]. Pineal region masses exemplify this scenario, as pre-operative CSF sampling is already implemented in clinical practice [59–61]. In our cohort, CNV detection enabled differentiation of benign and malignant pineal tumors. For clinical implementation at the time of diagnosis, a turnaround time of a few days in state-of-the-art pathology laboratories will be necessary. While the sensitivity of this approach needs to be prospectively evaluated, including additional tumor types and locations such as suprasellar masses, CNVs show high specificity for cancerous lesions. As the absence of CNVs in cfDNA profiles does not rule out malignancy, negative ctDNA results warrant short-interval follow-up by MRI and CSF assessment, potentially sparing high-risk neuro-surgical interventions after consideration of all clinical aspects by treating physicians. Moreover, chr12p gain, previously reported as an unfavorable prognostic factor in CNS GCTs [63], was detected in CSF samples of respective patients which may allow for therapeutic risk-stratification in the future.

For solid tumors including brain tumors, lcWGS pipelines can be developed as straight-forward assays for disease monitoring with a fast turnaround time akin to immunoglobulin G (IgG) and T-cell receptor (TCR) sequencing for MRD diagnostics in leukemia [21]. Consistent with previous studies [26, 27], our findings indicate that ctDNA detection by lcWGS was more sensitive during follow-up than CSF cytology, supporting the addition of molecular CSF pathology to routine cytological analysis. In patients with longitudinal CSF samples, cfDNA profiling has the potential to significantly enhance clinical management. When relapse was suspected or imaging results were unclear, distinct CNVs in cfDNA profiles confirmed relapse while simultaneously providing a molecular tumor profile and insights into tumor evolution. Liquid biopsies can also reduce the need for invasive tissue sampling and enable molecular tumor profiling in non-biopsiable cases, especially in challenging scenarios such as small relapsed tumor lesions or diffuse leptomeningeal disease. Conversely, ctDNA negative liquid biopsy results in the context of unclear MRI findings suggest a minimally invasive, short-interval surveillance strategy involving repeated MRI and CSF assessments, potentially avoiding unnecessary treatments. Although CSF samples in the current cohort were primarily collected at active disease or when disease was suspected, recent evidence from a large medulloblastoma trial indicated that lcWGS of CSF samples collected at regular routine follow-up visits may allow relapse detection prior to MRI [27]. Our data support the application of lcWGS for cfDNA profiling in prospective, multicenter trials with larger patient cohorts. Such studies are particularly warranted for entities with frequent CNVs, including embryonal tumors, ependymomas, pineoblastomas, and CNS GCTs, and will be instrumental in defining the clinical utility of lcWGS cfDNA assays.

Beyond binary tumor detection, our findings demonstrate the feasibility of retrieving insights into spatiotemporal heterogeneity and potential therapeutic targets from CSF during relapse, sometimes even reflecting early events not yet detectable from single tissue biopsies. This approach may also open avenues for systematically exploring therapy-induced tumor evolution and determining eligibility for trials based on cfDNA-derived CNVs, such as MYC(N) amplifications required for enrollment into the INFORM2 NivEnt trial [67].

While lcWGS has limited applicability for cases with copy number neutral tumors, such as most LGG/LGNTs, at diagnosis, it may reveal emergence of CNVs in evolving tumor genomes during follow-up. Given the low input requirements of this tumor detection method, additional assays may be performed on the same sample. To address the low detection rates in the group of LGG/LGNTs, characterized by low cell turnover and primarily driven by single alterations in the MAPK signaling pathway [68], more targeted approaches, including the detection of *BRAF V600E* mutations and *KIAA::BRAF* fusions, may be beneficial [25, 66, 69]. Furthermore, bioinformatics interrogation and integration of additional layers of cfDNA data derived from lcWGS data, such as fragment length or nucleosome occupancy [70, 71], may enhance monitoring of genetically silent tumors. Liquid biopsies also hold promise for managing high-grade gliomas, particularly diffuse midline gliomas (DMGs), where high-risk tumor locations limit safe tissue sampling, and sensitive longitudinal biomarkers may aid in therapeutic adjustments during rapid progression. These patients were underrepresented in our cohort (*n = *3) and only had serum liquid biopsies, all of which were negative by lcWGS. Previous studies have demonstrated the feasibility of detecting and tracking high-grade gliomas in the CSF, using lcWGS [25], ddPCR for H3K27M [28, 66], or panel sequencing [26].

While the sample size of our study limits definitive conclusions for individual entities or specific clinical questions, it provides a robust framework and methodology to prospectively tackle critical questions in the field of pediatric neuro-oncology: Which CSF sampling method and collection site serve as the most promising sources of ctDNA? What are the most informative time points concerning distinct clinical questions? Which tumor entities benefit from the integration of liquid biopsy analyses, and which do not? Are additional CSF draws beyond current standard-of-care justified for liquid biopsy profiling? Dedicated prospective direct comparisons of paired shunt and lumbar punctures, along with harmonized prospective trial implementation for assessing clinical utility, will be crucial next steps, as currently explored in the framework of the liquid biopsy working group of the European Society of Pediatric Oncology (SIOPE) brain tumor group. While these efforts present challenges, they also hold great promise. In the future, augmentation of frontline therapy, switch to salvage regimens, or therapy de-escalation may be guided by liquid biopsy, enabling more personalized and adaptive treatment strategies.

## Conclusions

In summary, we report the successful profiling of liquid biopsy samples without input limitations across a real-world institutional cohort of pediatric CNS tumor patients. Our sensitive lcWGS assay demonstrates clinical utility in use-cases across different tumor entities and diverse clinical scenarios. This provides the foundation for prospective validation of the clinical utility of liquid biopsies as they move toward clinical translation in pediatric neuro-oncology.

## Electronic supplementary material

Below is the link to the electronic supplementary material.

Supplementary Material 1

Supplementary Material 2

## Data Availability

The datasets used and/or analyzed during the current study are available from the corresponding author on reasonable request.

## Abbreviations

AFP: Alpha-fetoprotein
ATRT: Atypical teratoid/rhabdoid tumor
β-hCG: Beta human chorionic gonadotropin
cfDNA: Cell-free DNA
ddPCR: Droplet digital polymerase chain reaction
DMG: Diffuse midline glioma
CNS: Central nervous system
CSF: Cerebrospinal fluid
ctDNA: Circulating-tumor DNA
CNN: Copy number neutral
CNV: Copy number variation / variant
EPN: Ependymoma
EVD: External ventricular drain
GCT: Germ cell tumor
HGG: High-grade glioma
lcWGS: Low-coverage whole genome sequencing
LGG/LGNT: Low-grade glioma/ low-grade neuroepithelial tumor
LP: Lumbar puncture
MB: Medulloblastoma
MRI: Magnetic resonance imaging
MRD: Minimal residual disease
