# Supplementary material for: Ultra-low-input cell-free DNA sequencing for tumor detection and characterization in a real-world pediatric brain tumor cohort
Source: Acta Neuropathol Commun. 2025 Jun 28;13:134. doi: 10.1186/s40478-025-02024-w (PMC12205504; doi:10.1186/s40478-025-02024-w)
Supplement: Supplementary file 2 — Supplementary Material 2 [file 40478_2025_2024_MOESM2_ESM.docx]

**Supplementary materials**


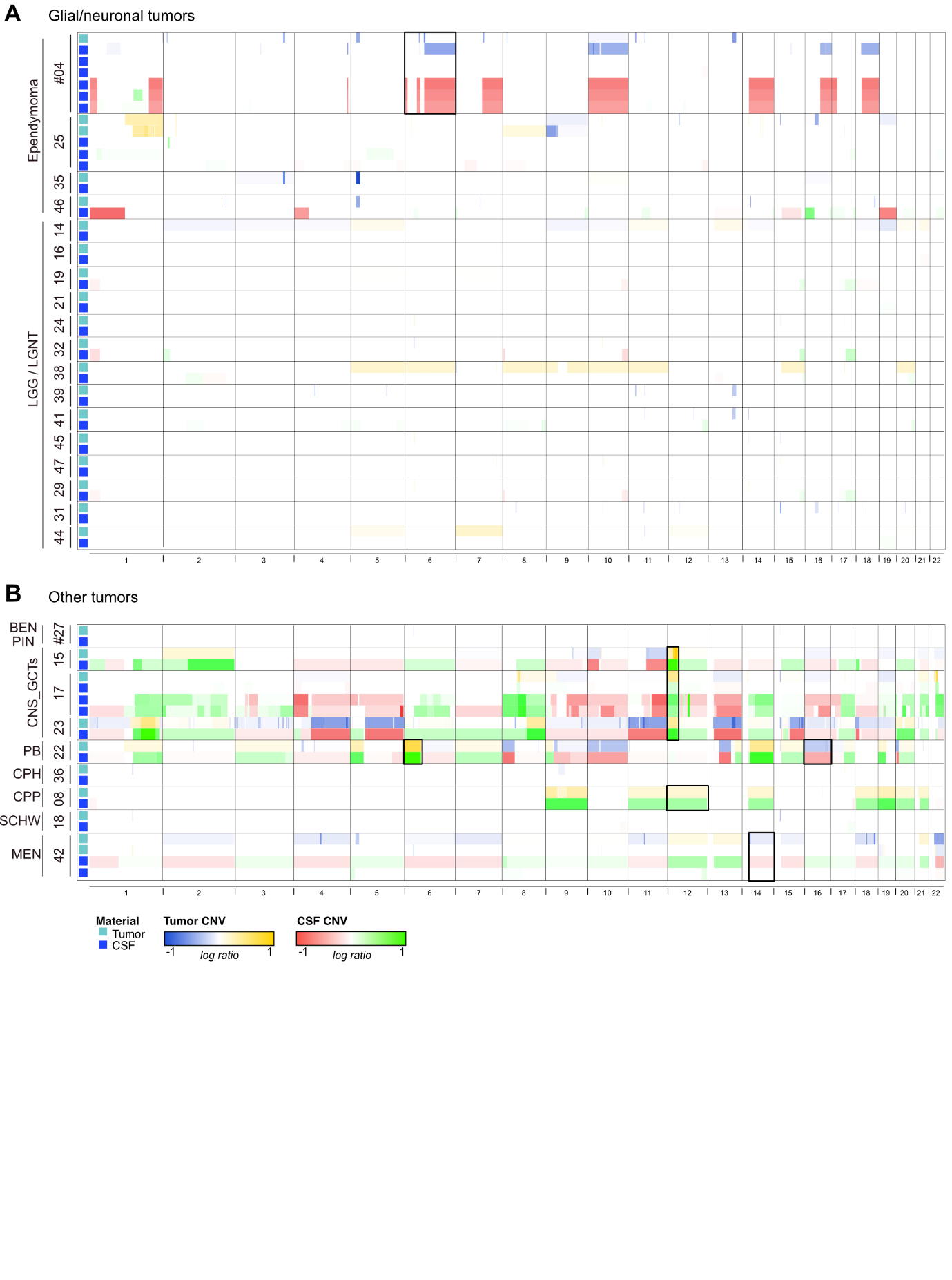


***Fig. S1***. **lcWGS-based tumor detection in pediatric CNS tumor liquid biopsies.** (**A**) Genome-wide CNV profiles of CSF and matched tumor samples for patients with glioneuronal CNS tumors. (**B**) Genome-wide CNV profiles of CSF and matched tumor samples for patients with other CNS tumors. Hallmark cytogenic events for each tumor entity are highlighted with black boxes.
*Abbreviations:* *BEN PIN = benign pineal tumor, CNS_GCTs = CNS germ cell tumors, PB = pineoblastoma, CPH = craniopharyngioma, CPP = choroid plexus papilloma, SCHW = schwannoma, MEN = meningioma.*

***
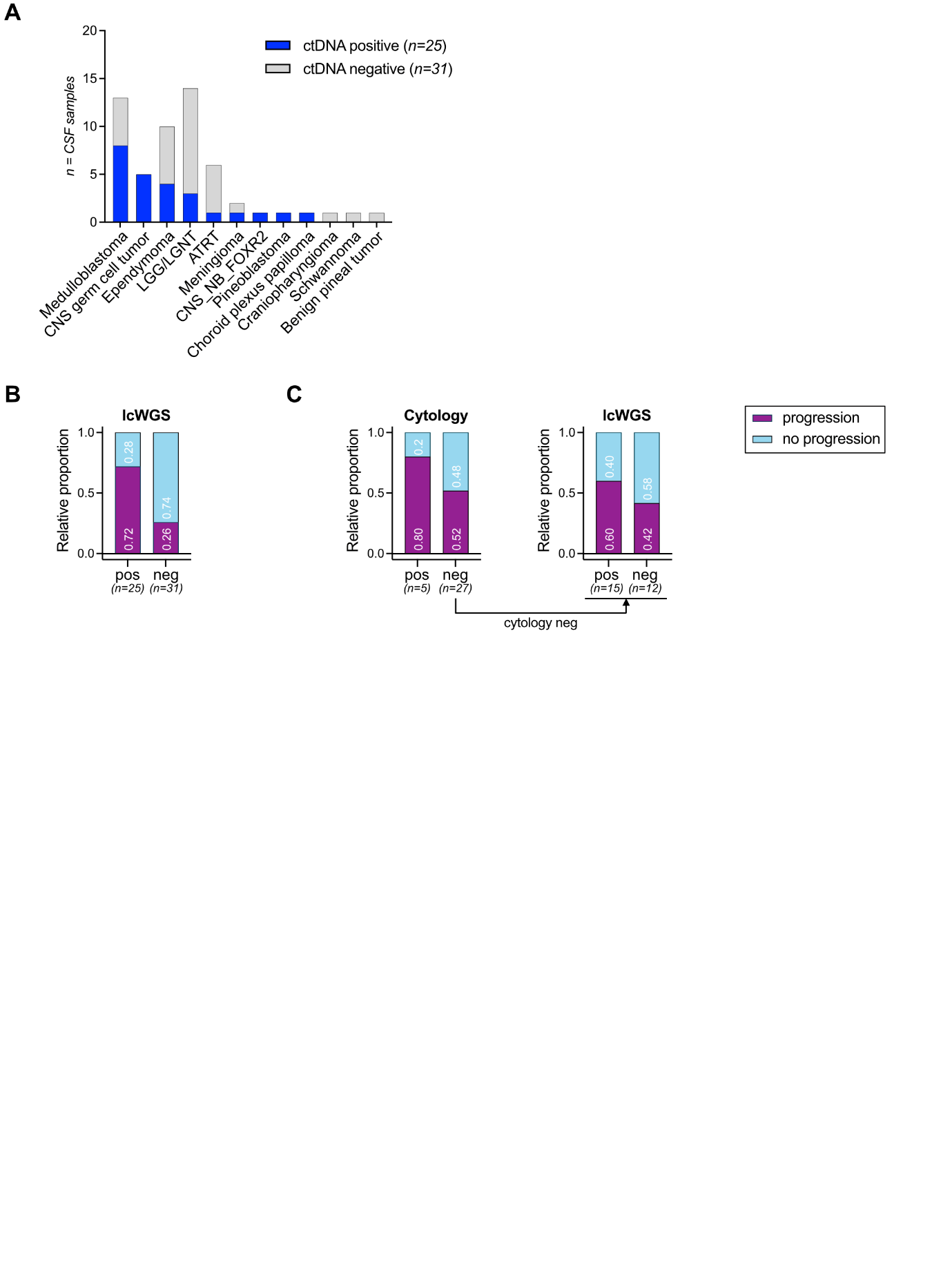
***

***Fig. S2***. **Detection of ctDNA across pediatric CNS tumor types and clinical scenarios**. (**A**) Ranked bar plot displaying number of ctDNA positive and ctDNA negative CSF samples per tumor entity in a cross-entity pediatric CNS tumor cohort (*n=36* patients, *n=56* CSF samples). (**B-C**) Stacked bar plots indicating progression rate as assessed by lcWGS and cytology analysis of CSF samples.


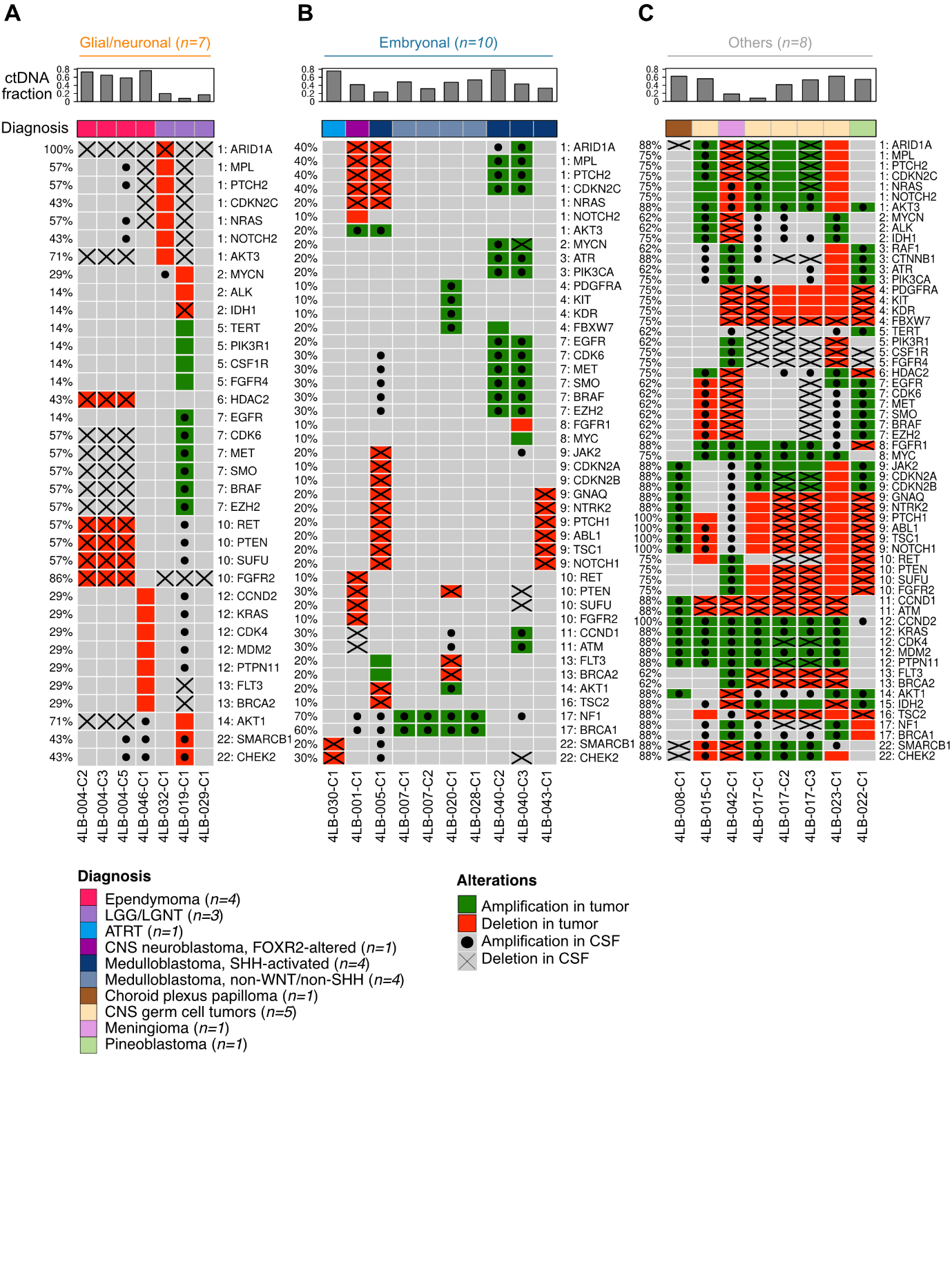


***Fig. S3***. **Annotation of CSF-derived cfDNA profiles with clinically reported target genes**. (**A-C**) CNVs in ctDNA positive CSF samples (*n=25*) were annotated with potentially actionable target genes according to the INFORM registry study, filtered for an established neuropathology panel to focus on CNS tumor targets [7, 9, 43-45].
